# Supplementary material for: Lactobacillus acidophilus Expressing Murine Rotavirus VP8 and Mucosal Adjuvants Induce Virus-Specific Immune Responses
Source: Vaccines (Basel). 2023 Nov 28;11(12):1774. doi: 10.3390/vaccines11121774 (PMC10747613; doi:10.3390/vaccines11121774)
Supplement: Supplementary file 1 [file vaccines-11-01774-s001.zip › Gilfillan-Vilander-etal_SupplementaryMaterials_Figures-Files.pdf]

Supplementary Figures

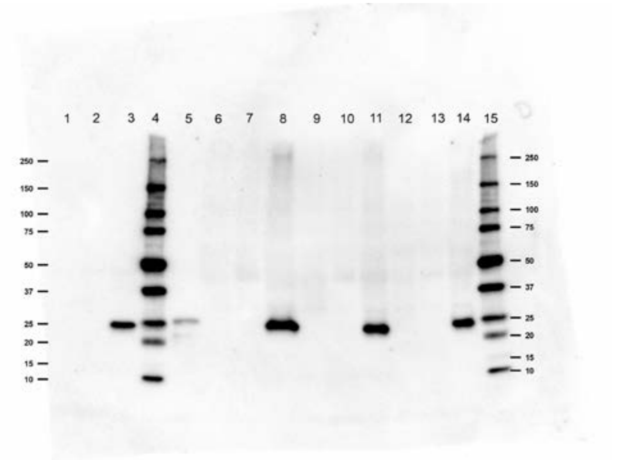

Figure S1. Uncropped western blot confirming VP8-1 rLA antigen cytoplasmic accumulation. Lanes 1: NCK56, 2: GAD80, 3: GAD85, 4: Precision Plus WesternC ladder (Bio-Rad, Hercules, CA, USA), 5: *E. coli* expressed VP8-1 positive control, 6: NCK56, 7: GAD80, 8: GAD85, 9: NCK56, 10: GAD80, 11: GAD85, 12: NCK56, 13: GAD80, 14: GAD85, 15: Precision Plus WesternC ladder (Bio-Rad).

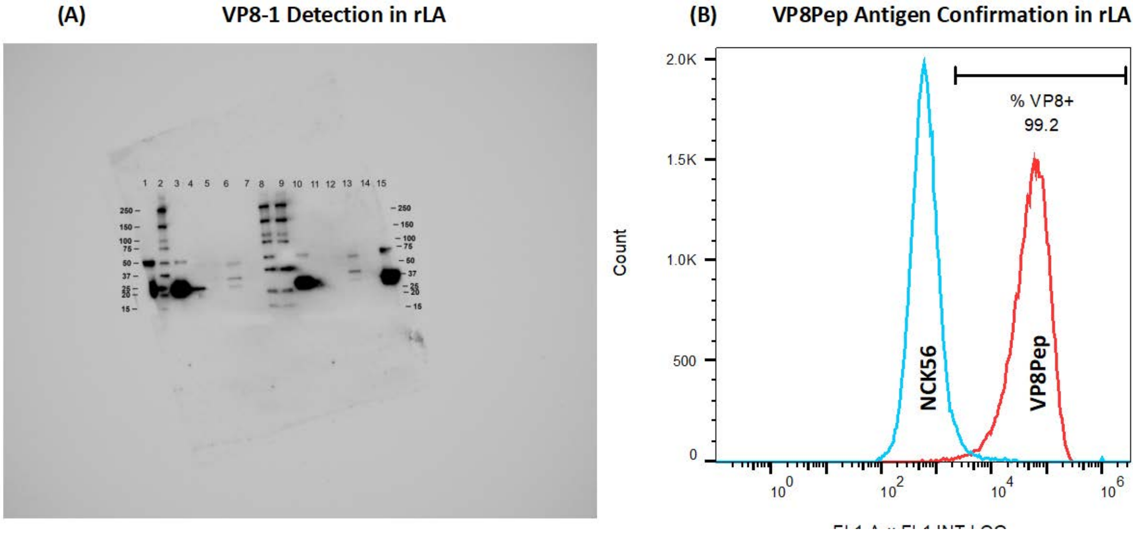

Figure S2. Uncropped western blot (A) confirming VP8-1 rLA antigen cytoplasmic accumulation in GAD84 with a 25 kDa band present in GAD84 lysates corresponding to the positive control. Western blot lanes 1: *E. coli* expressed VP8-1 positive control, 2: Precision Plus WesternC ladder (Bio-Rad), 3: *E. coli* expressed VP8-1 positive control, 4: GAD84, 5: NCK56, 6: GAD85, 7: NCK56, 8: Precision Plus WesternC ladder (Bio-Rad), 9: Precision Plus WesternC ladder (Bio-Rad), 10: *E. coli* expressed VP8-1 positive control, 11: GAD84, 12: NCK56, 13: GAD84, 14: NCK56, 15: *E. coli* expressed VP8-1 positive control. Flow cytometry histogram (B) confirming VP8Pep surface expression (red peak) in GAD84 as compared to the NCK56 negative control (blue peak).

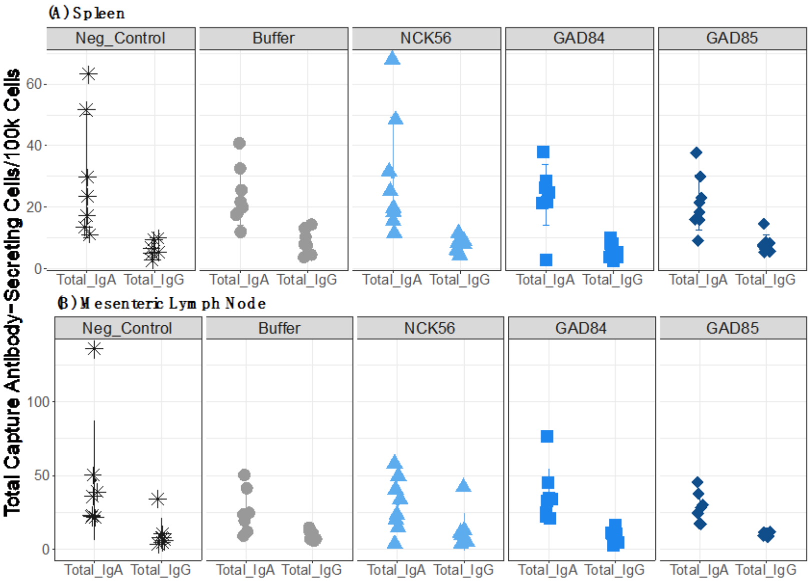

Figure S3. Total-capture IgA and IgG antibody secreting cells per 100,000 cells as measured by FluoroSpot in the spleen (A) and MLN (B) post oral immunization every other week with either control (negative control, Buffer, NCK56) or recombinant probiotic vaccine strains (GAD84, GAD85). MLN; mesenteric lymph node, VP8-1: full-length rotavirus protein antigen, VP8Pep; 10 amino-acid length rotavirus peptide antigen, rLA; recombinant *Lactobacillus acidophilus.*

Supplementary File S1. rLA VP8-1 and FimH Integration Methods.

The full length VP8-1 and FimH were integrated consecutively into the chromosome of *Lactobacillus acidophilus* (LA) strain GAD80 using the previously developed *upp*-based counter selective gene replacement system [1] and the previously constructed chromosomal integration plasmid pTRK1038 [2] that contains 600 bp of upstream and 600 bp of downstream sequences flanking the immediate downstream of the highly expressed gene *lba0889* (*enolase*). The FimH integration cassette containing the FimH N-terminal binding domain, PrtP signal peptide, and Mub anchor motif [3] was amplified from pTRKAV-04 [4] with primers NEB_fimH_fwd and NEB_fimH_rev. The amplified FimH cassette was assembled with NotI digested pTRK1030 backbone using NEBuilder HiFi DNA Assembly mix (New England BioLabs, NEB, Ipswich, MA, USA) to generate FimH integration plasmid pTRK1209. The reaction mixture was then heat-shock transformed into cloning host *E. coli* EC101 for amplification. The transformants were plated on BHI agar plates supplemented with 40 μg/ml kanmycin and 150 μg/ml erythromycin (Erm) and incubated at 30°C. Resulting colonies were screened with primers upp_fwd and upp_rev for positive transformants and confirmed by Sanger sequencing. Plasmid pTRK1209 was then electroporated into GAD80 that also contains plasmid pTRK669 (provides repA in trans for the replication of pTRK1209 [1]). Selection for single-crossover FimH recombinants was performed as previously described [1]. Briefly, single-crossover recombinants were selected after growing the GAD80 transformants at 42 °C for 48 hr in MRS supplemented with 2.5 μg/ml Erm. Single-crossover plasmid integration was confirmed by screening with primers repA-fwd and repA-rev. The single-crossover recombinants were then grown at 37 °C for 48 hr without antibiotic pressure, to facilitate the double-crossover recombination event. The double-crossover recombinants were selected with 100 μg/mL 5-fluorouracil (Sigma-Aldrich, St. Louis, MO, USA). The chromosomal integration (double-crossover) mutants were confirmed by PCR using primers 889_fwd and 889_rev. The resulting strain expressing the FimH cassette was designated LA NCK2753. The full length VP8-1 integration follows similar procedures described above. The VP8-1 was obtained as a gene block (IDT technologies, Coralville, IA, USA) with overlap sequences for downstream assembly. The VP8-1 integration plasmid was constructed by inserting the VP8-1 fragment and 660 bp of FimH flanking sequence (amplified from pTRK1209 with primers FimH_flanking_fwd and FimH_flanking_rev) into NotI and PvuI double-digested pTRK1038. The resulting VP8-1 integration plasmid pTRK1213 was transformed into NCK2753 with pTRK669. The procedures to select for chromosomal VP8-1 integration mutants were described as above. The resulting strain expressing both VP8-1 and FimH cassette downstream of *lba0889* was designated LA NCK2783.

1. Goh, Y.J.; Azcárate-Peril, M.A.; O'Flaherty, S.; Durmaz, E.; Valence, F.; Jardin, J.; Lortal, S.; Klaenhammer, T.R. Development and application of a upp-based counterselective gene replacement system for the study of the S-layer protein SlpX of Lactobacillus acidophilus NCFM. *Appl Environ Microbiol* **2009**, *75*, 3093-3105, doi:10.1128/aem.02502-08.
2. Douglas, G.L.; Klaenhammer, T.R. Directed chromosomal integration and expression of the reporter gene gusA3 in Lactobacillus acidophilus NCFM. *Appl Environ Microbiol* **2011**, *77*, 7365-7371, doi:10.1128/aem.06028-11.
3. Kajikawa, A.; Nordone, S.K.; Zhang, L.; Stoeker, L.L.; LaVoy, A.S.; Klaenhammer, T.R.; Dean, G.A. Dissimilar properties of two recombinant Lactobacillus acidophilus strains displaying Salmonella FliC with different anchoring motifs. *Appl Environ Microbiol* **2011**, *77*, 6587-6596, doi:10.1128/aem.05153-11.
4. Vilander, A.C.; Shelton, K.; LaVoy, A.; Dean, G.A. Expression of E. coli FimH Enhances Trafficking of an Orally Delivered Lactobacillus acidophilus Vaccine to Immune Inductive Sites via Antigen-Presenting Cells. *Vaccines* **2023**, *11*, 1162.

Supplementary File S2. Biorender.com publication license for Figure 1.

49 Spadina Ave. Suite 200
Toronto ON M5V 2J1 Canada
www.biorender.com

Confirmation of Publication and Licensing Rights

November 8th, 2023
Science Suite Inc.

Subscription:	*Institution*
Agreement number:	*EY262MEGB2*
Journal name:	*MDPI Vaccines*

To whom this may concern,

This document is to confirm that Darby Gilfillan has been granted a license to use the BioRender content, including icons, templates and other original artwork, appearing in the attached completed graphic pursuant to BioRender's Academic License Terms. This license permits BioRender content to be sublicensed for use in journal publications.

All rights and ownership of BioRender content are reserved by BioRender. All completed graphics must be accompanied by the following citation: "Created with BioRender.com".

BioRender content included in the completed graphic is not licensed for any commercial uses beyond publication in a journal. For any commercial use of this figure, users may, if allowed, recreate it in BioRender under an Industry BioRender Plan.

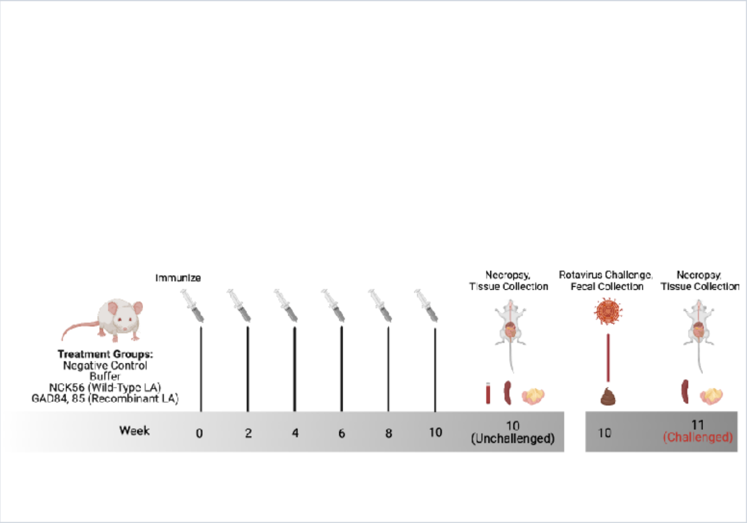

For any questions regarding this document, or other questions about publishing with BioRender refer to our BioRender Publication Guide, or contact BioRender Support at support@biorender.com.
